# Design and Activity Evaluation of Berberine-Loaded Dual pH and Enzyme-Sensitive Colon-Targeting Microparticles

**DOI:** 10.3390/pharmaceutics17060778

**Published:** 2025-06-13

**Authors:** Jingqi Sun, Xinlong Chai, Xiwen Zeng, Qingwei Wang, Yanwen Ling, Lihong Wang, Jin Su

**Affiliations:** 1 Department of Pharmaceutics, School of Pharmacy, China Jiamusi University, Jiamusi 154007, China; 238163038@stu.jmsu.edu.cn (J.S.); 228153054@stu.jmsu.edu.cn (X.C.); 23120640129@stu.jmsu.edu.cn (X.Z.); 13100997090@163.com (Q.W.); 228153056@stu.jmsu.edu.cn (Y.L.); wanglihong@jmsu.edu.cn (L.W.); 2 Key Laboratory of Biological Medicine Preparations of Heilongjiang Province, Jiamusi University, Jiamusi 154007, China

**Keywords:** berberine, colonic targeting, particles, ulcerative colitis, intestinal flora

## Abstract

Ulcerative colitis (UC) is a multifactorial disorder, and conventional oral berberine (BBR) suffers from poor colonic targeting. This study aimed to develop a colon-targeted microparticle system (BBR-ES MPs) based on chitosan (CS) and Eudragit S-100 to enhance BBR delivery efficiency and therapeutic efficacy in UC. **Methods**: BBR-CS nanocarriers were prepared via ionotropic gelation and coated with Eudragit S-100 to form pH/enzyme dual-responsive MPs. Colon-targeting performance was validated through in vitro release assays. SPF-grade male KM mice (Ethics Approval No.: JMSU-2021090301) with dextran sulfate sodium (DSS)-induced UC were divided into normal, model, BBR, and BBR-ES MPs groups. Therapeutic outcomes were evaluated by monitoring body weight, disease activity index (DAI), colon length, histopathology, inflammatory cytokines (IL-1β, IL-6, TNF-α, IL-10), and myeloperoxidase (MPO) activity via ELISA. Gut microbiota diversity was analyzed using 16S rRNA sequencing. **Results**: BBR-ES MP treatment significantly reduced DAI scores (*p* < 0.01), restored colon length, downregulated pro-inflammatory cytokines (IL-1β, IL-6, TNF-α; *p* < 0.05), and upregulated anti-inflammatory IL-10. Microbiota analysis revealed that the Bacteroidetes/Firmicutes ratio, which decreased in the model group, was restored post-treatment, with alpha/beta diversity approaching normal levels. BBR-ES MPs outperformed free BBR at equivalent doses. **Conclusion**: BBR-ES MPs achieved colon-targeted drug delivery via pH/enzyme dual-responsive mechanisms, effectively alleviating UC inflammation and modulating gut dysbiosis, offering a safe and precise therapeutic strategy for UC management.

## 1. Introduction

Epidemiological data indicate that ulcerative colitis (UC) has emerged as a globally prevalent disease [1]. In North America, the prevalence of UC ranges from 37.5 to 248.6 per 100,000 individuals, while in Europe, it varies between 4.9 and 505 per 100,000 [2]. Evidence suggests that in China, UC incidence rates are rising due to population aging, environmental challenges, and other factors. Although the exact etiology of UC remains unclear, recent studies propose associations with environmental triggers, nutritional imbalances, immune dysregulation, and genetic predisposition [3].

Berberine (BBR), an isoquinoline alkaloid, appears as an orange or yellow powder with a bitter taste and faint odor [3,4]. It exhibits diverse pharmacological activities, including anti-inflammatory, antimicrobial [5], cardioprotective [6], antitumor [7,8], and hypoglycemic effects [9]. However, its high polarity limits gastrointestinal absorption due to rapid adsorption.

Oral colon-specific drug delivery systems (OCDDS), developed in the 1980s, enable targeted drug release in the colon while preventing premature release in the upper gastrointestinal tract (GIT). Coated tablets, capsules, and pellets protect drugs from gastric pH/enzymes, enhance stability, reduce adverse effects, and improve bioavailability by forming uniform dispersions in the intestine, enabling therapeutic efficacy at lower doses [10,11]. However, conventional oral colon-targeted drug delivery systems (OCDDS) are constrained by limitations such as unreliable targeting efficiency, interpatient variability, and suboptimal solubility profiles for specific therapeutics. In contrast, nanoengineered OCDDS leverage nanocarriers or advanced nanoformulations to amplify colonic drug uptake through synergistic mechanisms, including macrophage-mediated phagocytosis, enhanced epithelial permeability, and mucoadhesive retention, thereby prolonging therapeutic exposure at disease sites [12].

In this study, we designed a dual pH- and enzyme-responsive colon-targeting microparticle system for UC treatment. Berberine-loaded chitosan nanoparticles (CS NPs) were first prepared via ionic gelation to confer enzyme sensitivity, followed by coating with Eudragit S-100 using emulsion-solvent evaporation to achieve micron-scale particles with pH responsiveness. The physicochemical properties and drug release profiles were characterized. A dextran sulfate sodium (DSS)-induced UC mouse model was established to evaluate targeting efficiency, tissue distribution, and therapeutic outcomes. Metrics included disease activity index (DAI) scores, colon length, histopathology, myeloperoxidase (MPO) activity, inflammatory cytokine levels (IL-1β, IL-6, TNF-α, IL-10), and gut microbiota composition via high-throughput sequencing.

Results demonstrated that BBR-ES MPs significantly alleviated UC symptoms, reduced inflammatory markers, and restored gut microbiota diversity. The dual-responsive system ensured targeted colonic release, enhancing therapeutic efficacy at lower doses while minimizing systemic exposure. This study provides a mechanistic foundation for BBR-based UC therapy and advances the development of precision-targeted nanomedicines for inflammatory bowel diseases.

## 2. Materials and Methods

### 2.1. Materials

Berberine (98%), the DiR cell membrane fluorescent probe, dextran sodium sulfate (DSS) and β-glucosidase were purchased from Dalian Meilun Biotech Co., Ltd., Dalian, China. Chitosan and sodium tripolyphosphate (TPP) were purchased from Shanghai Yuanye Biotechnology Co., Ltd., Shanghai, China. Glacial acetic acid, Span-80, liquid paraffin, petroleum ether, and phosphoric acid were purchased from Tianjin Komeo Chemical Reagent Co, Ltd., Tianjin, China. ELISA kits were purchased from Shanghai Enzymolink Bio-technology Co, Ltd., Shanghai, China. Mesalazine was purchased from Jiamusi Luling Pharmaceutical Co., Ltd., Jiamusi, China. A Fast DNA SPIN Kit was purchased from MP Biomedicals (Santa Ana, CA, USA), a PicoGreen dsDNA Analysis Kit was purchased from Invitrogen (Carlsbad, CA, USA), and a NovaSeq 6000 SP Reagent Kit was purchased from Wuhan Frasergen Bioinformatics Co., Ltd., Wuhan, China.

### 2.2. Preparation of Berberine Chitosan Nanoparticles (BBR-CS NPs)

To prepare BBR-CS NPs, 100 mg of chitosan (CS) was dissolved in 100 mL of 1% acetic acid to form a 1 mg·mL^−1^ CS solution, followed by the addition of berberine (BBR) and pH adjustment to 4.5 using 2 mol·L^−1^ sodium hydroxide. A separate 1 mg·mL^−1^ tripolyphosphate (TPP) solution in ultrapure water was prepared and added dropwise to the continuously stirred CS solution until faint blue opalescence appeared, yielding the nanoparticles [12]. The particle size and distribution of the BBR-CS NPs were analyzed using a laser particle size analyzer. Single-factor formulation optimization (results in Tables S1–S8 and Figures S1 and S2) identified the optimal parameters: a CS:TPP mass ratio of 3.3:1, a drug-loading ratio of 5.2:1, a CS concentration of 1 mg·mL^−1^, a stirring speed of 600 rpm, and a stirring duration of 11.8 min. For TEM imaging, a BBR-CS NP solution was diluted to an appropriate concentration, deposited onto a carbon-coated copper grid, air-dried at room temperature, and observed under Transmission Electron Microscopy (TEM) to assess morphology and size.

### 2.3. Preparation of Berberine Microparticles (BBR-ES MPs)

The prepared BBR-CS NPs were transferred into vials, pre-frozen at −20 °C for 12 h, and lyophilized to obtain a BBR-CS NP freeze-dried powder. Microparticles were fabricated using the emulsion-solvent evaporation method, with formulation screening results detailed in Tables S9 and S10. Eudragit S-100 and the BBR-CS NP freeze-dried powder were mixed at a 3:1 mass ratio, blended with 3 mL of anhydrous ethanol under stirring, and supplemented with additional Eudragit S-100 to form a homogeneous suspension. This suspension was added dropwise into 50 mL of liquid paraffin containing 1% Span-80, stirred at 40 °C for 3 h to evaporate ethanol, and then filtered under vacuum. The collected particles were washed with petroleum ether to remove residual paraffin and dried at 50 °C for 3 h, yielding yellow berberine-loaded microparticles (BBR-ES MPs) [13,14].

### 2.4. Characterization of BBR-CS NPs and BBR-ES MPs

The BBR-CS NP and BBR-ES MP samples were dried in a desiccator, fixed onto stubs using conductive carbon tape, and sputter-coated with gold to enhance conductivity. Morphological analysis was performed using scanning electron microscopy (SEM) at an accelerating voltage of 1–5 kV. For Fourier-transform infrared (FTIR) spectroscopy, samples including BBR raw material, CS, Eudragit S-100, a physical mixture of BBR and CS (1:5.2 mass ratio), CS NPs, BBR-CS NPs, and BBR-ES MPs were prepared. Each sample was thoroughly mixed with 100 mg of potassium bromide (KBr), ground into fine powder, pressed into pellets, and scanned over a wavenumber range of 4000–500 cm^−1^.

To simulate gastrointestinal drug release, BBR, BBR-CS NPs and BBR-ES MPs (containing equivalent BBR doses based on drug-loading capacity) were placed into dialysis bags (MWCO = 3500 Da, Beijing Sanyan Technology Co., Ltd.) containing 440 mL of release media: pH 1.2 HCl (0–2 h), pH 6.8 PBS (2–6 h), and pH 7.4 PBS (6–24 h). The bags were incubated at 37 °C with shaking at 100 rpm. At 6 h, BBR-ES MPs were further divided into pH 7.4 PBS with or without β-glucosidase (β-G). At predetermined intervals, 2 mL of medium was withdrawn and replaced with fresh medium. BBR concentration was quantified via HPLC (Agilent ZORBAX Eclipse XDB-C18 column, 4.6 × 250 mm, 5 μm; mobile phase: acetonitrile-0.2% phosphoric acid aqueous solution, 30:70 *v/v*; column temperature: 25 °C; detection wavelength: 349 nm; flow rate: 1 mL/min; injection volume: 10 μL). Cumulative drug release percentages were calculated [15].

### 2.5. Detection of Encapsulation Rate and Drug Loading

The encapsulation efficiency (EE) was determined indirectly [16]. Briefly, 5 mg of microparticles were placed in a 5 mL centrifuge tube, mixed with 5 mL of deionized water, and centrifuged at 12,000 rpm for 15 min at 4 °C. The supernatant containing the unencapsulated (free) drug was collected and filtered through a microporous membrane. The drug content in the supernatant was quantified using the HPLC method described in Section 2.

For drug loading (DL), the direct method was employed [16]. After centrifugation, the residual solution was removed, and the microparticles were dissolved in 10 mL of methanol under constant shaking at 37 °C for 24 h, followed by 30 min of ultrasonication to ensure complete drug release. The solution was filtered through a microporous membrane to obtain the encapsulated drug. EE and DL were calculated using the following equations:

EE (%) = (Total drug − Free drug)/Total drug × 100%(1)

DL (%) = Encapsulated drug/Total weight of microparticles × 100% (2)

### 2.6. In Vivo Targeting of Berberine Particles

To investigate the gastrointestinal tract (GIT) distribution of BBR-CS NPs and BBR-ES MPs in mice, infrared dye DiR was loaded into NPs and MPs as a substitute for BBR. DIR-CS NPs were prepared following the method described in Section 2.2 and freeze-dried, while DIR-ES MPs were prepared according to the protocol in Section 2.3; 14 SPF-grade male KM mice (weight 18–22 g, supplied by Changchun Yisi Laboratory Animal Technology Co., Ltd., Changchun, China) License No.: SYXK (Hei)-2021-018) were used. All animal experiments strictly complied with the Experimental Animal Management Regulations of Jiamusi University and were approved by the Institutional Animal Care and Use Committee of Jiamusi University (Ethics Approval No.: JMSU-2021090301). Mice were sacrificed at 6 h and 12 h post-administration (three mice per time point for experimental groups, one mouse per time point for the blank control group). The entire GIT was excised and imaged using IVIS Spectrum system with 720 nm excitation and 790 nm emission wavelengths.

### 2.7. The Therapeutic Effect of Berberine Microparticles on UC

#### 2.7.1. Animal Grouping

Sixty SPF-grade male KM mice were housed under standard conditions with free access to food and water. After a 7-day acclimation period (bedding replaced every two days), all mice were weighed and randomly divided into six groups (10 mice per group) based on comparable body weights: Control group (Ctrl), Colitis model group (Colitis), Mesalazine-positive group (MS), Berberine bulk drug group (BBR), Berberine chitosan nanoparticle group (CS NPs), and Berberine microparticle group (ES MPs). All treatment groups received the same oral dose of 50 mg·kg^−1^ via gavage administration.

#### 2.7.2. Establishment and Administration of UC Model

A 3% (*w/v*) dextran sulfate sodium (DSS) solution was prepared by dissolving DSS in distilled water [17]. Male KM mice were allowed free access to the DSS solution for seven consecutive days to induce colitis, with daily monitoring of DSS consumption, body weight changes, and fecal consistency (e.g., diarrhea, bloody stools). After the acclimatization period, all groups except the normal control (Ctrl) underwent DSS induction, while Ctrl mice received distilled water. During the seven-day modeling phase, body weight and fecal status were recorded daily. Post-modeling, therapeutic interventions were initiated: treatment groups (MS, BBR, CS NPs, ES MPs) received oral gavage of corresponding drugs at 50 mg/kg, whereas the Ctrl and Colitis groups were administered equivalent volumes of distilled water for seven days. Body weight and fecal parameters were continuously monitored throughout the treatment period.

#### 2.7.3. Disease Activity Index (DAI) Evaluation

During both the modeling and treatment periods, the disease activity index (DAI) was assessed daily to monitor colitis progression in mice. The DAI score incorporates three parameters: weight loss, stool consistency, and rectal bleeding, each graded on a 0–4 scale. As detailed in Table 1, weight loss was scored as 0 (no loss), 1 (1–5% loss), 2 (6–10% loss), 3 (11–15% loss), or 4 (>15% loss). Stool consistency was graded as 0 (normal), 2 (loose stools), or 4 (watery stools), while rectal bleeding was scored as 0 (none), 2 (mild bleeding), or 4 (severe bleeding). The clinical score, calculated as the average of these three scores, ranged from 0 (healthy) to 4 (maximal colitis). The percentage of weight change was determined using the following formula [18]:

Weight change (%) = Initial weight − Current weight × 100%(3)

#### 2.7.4. Animal Sacrifice and Tissue Preservation

After the final drug administration, mice were fasted for 24 h, euthanized by cervical dislocation, and the colon (from cecum to distal end) was excised. The colon was rinsed with saline, and a 1–2 cm segment was homogenized in ice-cold PBS (pH 7.4, 9 × volume) using a tissue homogenizer. The homogenate was centrifuged (4 °C, 4000 rpm, 15 min), and the supernatant was stored at −80 °C for subsequent analysis. Fecal pellets (1–2 per mouse) were collected and flash-frozen in liquid nitrogen.

#### 2.7.5. Colon Length Measurement

The excised colon was laid flat on white paper, and its length (from cecum to distal end) was measured using a ruler. Mean colon length was calculated for each group.

#### 2.7.6. Histopathological Examination

Colon tissues were rinsed with saline, longitudinally incised, and fixed in 4% paraformaldehyde. After paraffin embedding and sectioning, hematoxylin and eosin (HE) staining was performed to evaluate pathological changes under a light microscope.

#### 2.7.7. Myeloperoxidase (MPO) Activity Assay

MPO activity in colon homogenates was quantified using a commercial ELISA kit. Briefly, 200 μL of thawed supernatant was transferred to a 1.5 mL microcentrifuge tube, and MPO levels were measured according to the manufacturer’s protocol [19].

#### 2.7.8. Inflammatory Cytokine Analysis

Levels of IL-1β, IL-6, IL-10, and TNF-α in colon homogenates were determined via ELISA. Aliquots (200 μL) of supernatant were analyzed using cytokine-specific kits following the manufacturer’s instructions [20].

### 2.8. Impact of Berberine Microparticles on the Gut Microbiota in UC Mice

To investigate the mechanism by which berberine microparticles ameliorate UC through modulation of the gut microbiota, fecal DNA was extracted and subjected to high-throughput sequencing on the Illumina NovaSeq platform. Alpha diversity, beta diversity, taxonomic composition, and differential species analyses were performed to assess microbial shifts induced by DSS and BBR-ES MPs intervention, providing insights into the pharmacological mechanisms of BBR [21].

#### 2.8.1. Fecal Sample Collection and Sequencing

Fecal samples from three randomly selected mice per group were collected for sequencing.

#### 2.8.2. Bioinformatics and Taxonomic Profiling

Paired-end sequencing was conducted using the Illumina NovaSeq platform. Raw sequences were processed via the QIIME2 dada2 pipeline for denoising, generating amplicon sequence variants (ASVs). Taxonomic classification was performed against the Greengenes database (16S rRNA reference). ASV/OTU tables were rarefied to 95% of the minimum sample depth to standardize sequencing depth. Taxonomic composition was visualized at multiple classification levels [22].

#### 2.8.3. Alpha Diversity Analysis

Alpha diversity indices (Chao1, Observed species, Shannon, Simpson) were calculated using QIIME2 to evaluate microbial richness and evenness. Rank-abundance curves were plotted with log2-transformed abundance values using RadaR Language software [23].

#### 2.8.4. Beta Diversity Analysis

Beta diversity was assessed using rarefied ASV/OTU tables. Principal coordinate analysis (PCoA) based on Bray-Curtis distances and non-metric multidimensional scaling (NMDS) were performed to visualize inter-group microbial community differences. Statistical significance was tested via permutational multivariate analysis of variance (PERMANOVA) [24].

#### 2.8.5. Differential Species and Biomarker Identification

Venn diagrams, heatmaps of taxonomic composition, and orthogonal partial least squares-discriminant analysis (OPLS-DA) were generated using RadaR to identify differentially abundant taxa and microbial biomarkers across groups [25].

### 2.9. Statistical Analysis

Data are expressed as mean ± standard deviation (SD) from at least three independent experiments. One-way ANOVA followed by post hoc tests was performed using SPSS 21.0. Significance levels were denoted as * *p* < 0.05, ** *p* < 0.01, *** *p* < 0.001, **** *p* < 0.0001, # *p* < 0.05, ## *p* < 0.01, ### *p* < 0.001, #### *p* < 0.0001, with “NS” indicating non-significance.

## 3. Results

### 3.1. Synthesis and Characterization of BBR-CS NPs

**Figure 1:**
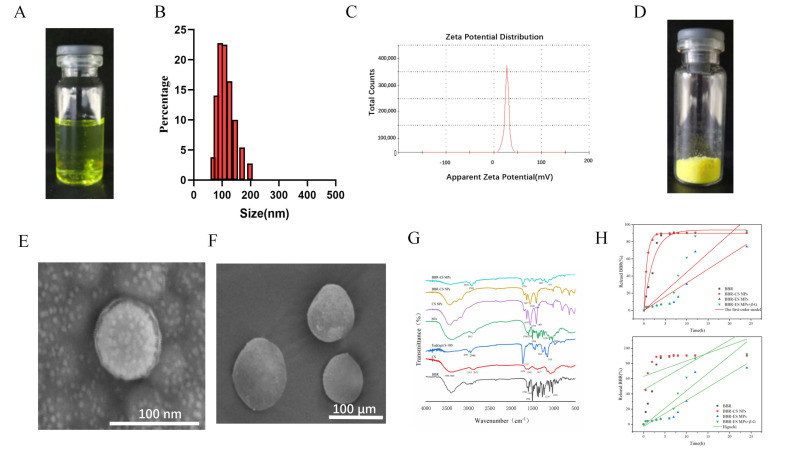

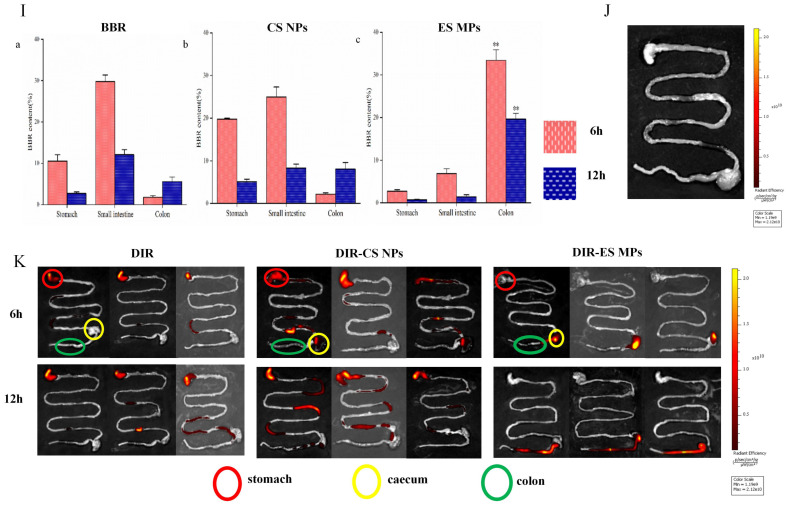
Characterization of BBR-CS NPs and BBR-ES MPs, and in vivo targeting evaluation (**A**) Macroscopic view of BBR-CS NPs. (**B**,**C**) Particle size distribution and zeta potential of BBR-CS NPs. (**D**) Lyophilized powder of BBR-ES MPs. (**E**) SEM image of BBR-CS NPs. (**F**) SEM image of BBR-ES MPs. (**G**) FTIR analysis of BBR, CS, Eudragit S-100, physical mixture, CS NPs, BBR-CS NPs, and BBR-ES MPs. (**H**) In vitro release of the first-order model and Higuchi model (*n* = 3). (**I**) Percentage content of BBR in the gastrointestinal tract (stomach, small intestine, and colon including cecum) at 6 h and 12 h post-administration of (**a**) BBR, (**b**) CS NPs, and (**c**) ES MPs (*n* = 3). ** *p* < 0.01. (**J**,**K**) Biodistribution of blank NPs, DiR, DiR-CS NPs, and DiR-ES MPs along the gastrointestinal tract.

Berberine-loaded chitosan nanoparticles (BBR-CS NPs) were synthesized via ionic gelation, yielding a yellow, transparent solution (Figure 1A). Laser particle size analysis revealed a uniform monomodal distribution with an average hydrodynamic diameter of 140.75 ± 0.86 nm and a zeta potential of 35.42 ± 0.43 mV (Figure 1B,C), indicating excellent colloidal stability. The encapsulation efficiency (EE) and drug loading (DL) of BBR-CS NPs were 89.15 ± 0.79% and 10.6 ± 0.51%, respectively.

### 3.2. Synthesis and Characterization of BBR-ES MPs

BBR-ES MPs were prepared using emulsion-solvent evaporation. The freeze-dried BBR-CS NPs appeared as a yellow flaky powder (Figure 1D), while BBR-ES MPs formed yellow spherical microparticles with smooth surfaces (Figure 1E), confirming successful coating with Eudragit S-100. BBR-ES MPs exhibited an EE of 74.25 ± 2.74% and DL of 2.56 ± 0.43%. SEM images (Figure 1E,F) demonstrated that BBR-CS NPs were spherical nanoparticles, whereas BBR-ES MPs exhibited larger, uniformly coated microparticles.

Figure 1G displays the FTIR spectra of BBR, CS, Eudragit S-100, a physical mixture of BBR and CS, CS NPs, BBR-CS NPs, and BBR-ES MPs. In the BBR spectrum, characteristic peaks included aromatic skeletal vibrations at 1598 cm^−1^ and 1504 cm^−1^, C–N stretching at 1386 cm^−1^, and C–O–C stretching at 1230 cm^−1^ and 1035 cm^−1^. The CS spectrum showed broad O–H/N–H stretching (3500–3000 cm^−1^), C–H stretching (2913 cm^−1^ and 2871 cm^−1^), and amide bands (amide I at 1658 cm^−1^, amide II at 1592 cm^−1^, and amide III at 1417 cm^−1^). Eudragit S-100 exhibited peaks for C–H stretching (2991 cm^−1^ and 2946 cm^−1^), C=O stretching (1727 cm^−1^), and C–O stretching (1263 cm^−1^ and 1155 cm^−1^). The physical mixture retained peaks of both BBR and CS, confirming simple superposition. In CS NPs, the amide I band shifted from 1658 cm^−1^ to 1637 cm^−1^, and the amide II band shifted to 1554 cm^−1^, indicating ionic crosslinking between protonated CS (–NH_3_^+^) and TPP phosphate groups. BBR-CS NPs showed no BBR-specific peaks, confirming encapsulation via CS-TPP crosslinking. Similarly, BBR-ES MPs aligned spectrally with Eudragit S-100, with no detectable BBR peaks, verifying dual encapsulation.

Figure 1H illustrates the drug release profiles under simulated gastrointestinal conditions. BBR exhibited rapid release (43.4% at 2 h, 88.9% at 6 h). BBR-CS NPs showed acid-triggered burst release (82.1% at 2 h) due to CS dissolution in gastric pH, with 89.8% cumulative release at 6 h. In contrast, BBR-ES MPs released only 7.6% within 6 h, followed by pH/enzyme-dependent release in colonic conditions (pH 7.4 PBS + β-glucosidase), achieving 92.5% cumulative release at 24 h. This dual-responsive behavior arose from Eudragit S-100 dissolution at neutral pH and enzymatic degradation of CS by β-glucosidase, enabling colon-specific drug release. The combined pH-enzyme response prevented premature drug release in the upper gastrointestinal tract while ensuring sustained and enhanced drug delivery in the colon, thereby optimizing therapeutic efficacy. The equations and parameters of the first-order model and HIGUCHI model are listed in Table 2.

### 3.3. Tissue Distribution Test Results

Figure 1I illustrates the quantitative distribution of BBR in the gastrointestinal tract (GIT) of colitis mice. At 6 h post-administration, the BBR and CS NP groups exhibited the highest BBR levels in the stomach and small intestine, which decreased by 12 h, while the ES MP group showed minimal BBR in these regions. In contrast, BBR accumulation in the colon (including the cecum) was significantly higher in the ES MP group compared to the BBR and CS NP groups at both 6 h and 12 h (*p* < 0.01). Combined with in vitro release data and ex vivo imaging, these results confirm that dual pH- and enzyme-responsive MPs enhance colonic targeting compared to raw BBR and single-response formulations.

Fluorescence imaging of DiR-labeled formulations (Figure 1J,K) revealed distinct biodistribution patterns. Blank NPs showed no fluorescence, confirming no interference from the carrier. DiR and DiR-CS NPs exhibited strong gastric and small intestinal signals at 6 h, with residual fluorescence in the upper GIT at 12 h, indicating rapid release and systemic absorption. In contrast, DiR-ES MPs showed no upper GIT fluorescence at 6 h but prominent signals in the distal ileum and proximal colon, which intensified in the colon by 12 h. This demonstrates that Eudragit S-100 effectively prevented premature drug release in the upper GIT, while enzymatic degradation in the colon facilitated targeted drug accumulation in inflamed tissues, validating the dual-responsive design’s efficacy.

### 3.4. Therapeutic Efficacy of Berberine Microparticles in UC Mice

#### 3.4.1. Body Weight Changes

**Figure 2:**
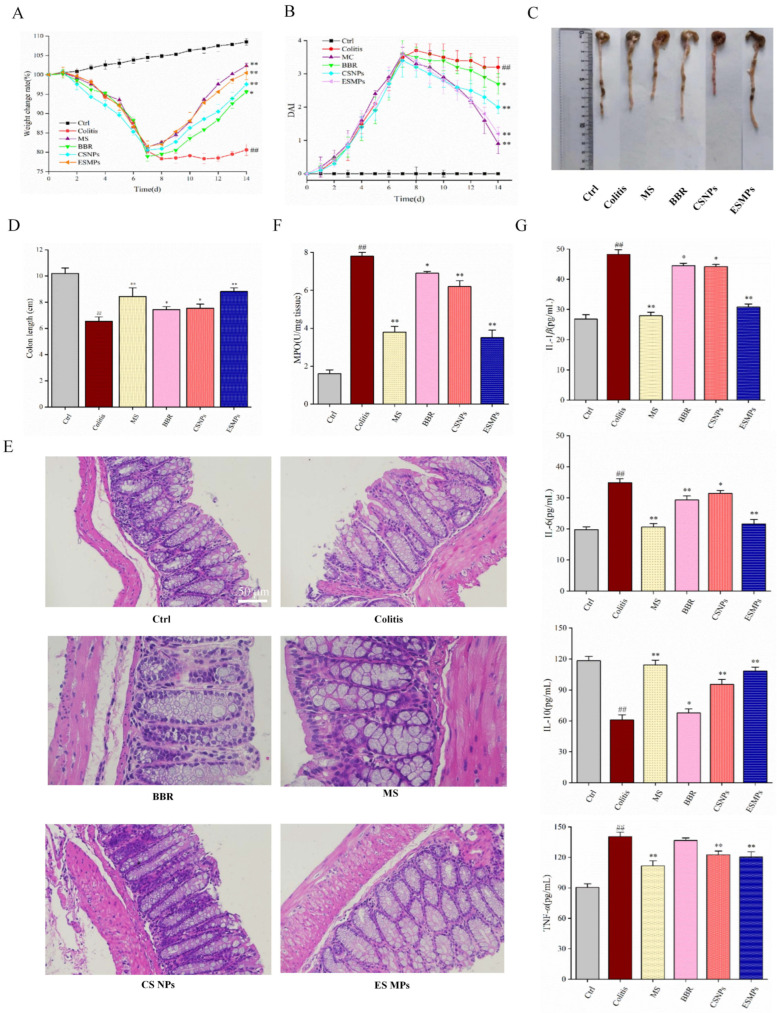
Therapeutic effects of berberine microparticles in UC mice (Significance levels were denoted as * *p* < 0.05, ** *p* < 0.01, ## *p* < 0.01). (**A**) Body weight changes (*n* = 10). (**B**) Daily disease activity index (DAI) scores (*n* = 10). (**C**,**D**) Macroscopic colon appearance and length measurements (*n* = 10). (**E**) Histopathological images of colon tissues (H&E staining). (**F**) Myeloperoxidase (MPO) activity (*n* = 7). (**G**) Inflammatory cytokine levels in colon tissues (*n* = 7).

As shown in Figure 2A, mice in all groups exhibited normal fecal consistency and robust health prior to DSS induction. During modeling, mice in non-control groups began losing weight by Day 2, with an average 20% reduction by Day 7. Post-treatment, weight recovery was observed in all therapeutic groups, particularly in the ES MP and mesalazine (MS) groups (*p* < 0.01). The Colitis group showed no significant recovery, while the normal Ctrl group exhibited steady weight gain over 14 days. These results indicate that BBR-based treatments alleviated DSS-induced weight loss, with ES MPs and MS demonstrating superior efficacy.

#### 3.4.2. Disease Activity Index (DAI) Scores

Figure 2B shows that Ctrl mice maintained stable DAI scores (0–0.5), whereas the Colitis group exhibited progressively worsening scores (up to 3.8 by Day 7), confirming successful UC induction. Therapeutic interventions significantly reduced DAI scores, with the ES MP and MS groups showing the most pronounced reductions (*p* < 0.01). Dual pH/enzyme-responsive MPs outperformed raw BBR and enzyme-sensitive MPs, highlighting their enhanced therapeutic potential.

#### 3.4.3. Measurement Results of Colon Length

Colon shortening and congestion were severe in the Colitis group compared to Ctrl (*p* < 0.01; Figure 2C,D). Treatment groups exhibited partial restoration of colon length, with MS and ES MPs achieving near-normal lengths (*p* < 0.01), demonstrating the efficacy of BBR-loaded MPs in mitigating DSS-induced structural damage.

#### 3.4.4. Histopathological Analysis

Histopathological evaluation (Figure 2E) revealed intact epithelial layers and normal glandular structures in Ctrl mice. The Colitis group displayed severe mucosal erosion, neutrophil infiltration, and gland loss. The BBR and CS NP groups showed moderate improvement, while the MS and ES MP groups exhibited near-normal mucosal architecture, minimal inflammation, and restored epithelial integrity, confirming their potent anti-inflammatory effects.

#### 3.4.5. Myeloperoxidase (MPO) Activity

MPO activity, a marker of neutrophil infiltration, was significantly elevated in the Colitis group (*p* < 0.01; Figure 2F). Treatment reduced MPO levels, with the MS, CS NP, and ES MP groups showing the most substantial declines (*p* < 0.01), indicating targeted suppression of neutrophil-driven inflammation.

#### 3.4.6. Inflammatory Cytokine Levels

Pro-inflammatory cytokines (IL-1β, IL-6, TNF-α) were markedly elevated in the Colitis group, while anti-inflammatory IL-10 was suppressed (*p* < 0.01; Figure 2G). Therapeutic interventions significantly lowered IL-1β, IL-6, and TNF-α (*p* < 0.01 or *p* < 0.05) and elevated IL-10 (*p* < 0.01), with the MS and ES MP groups exhibiting the strongest modulation. Notably, raw BBR had no significant effect on TNF-α (*p* > 0.05), underscoring the advantage of colon-targeted MPs.

### 3.5. Effects of Berberine Microparticles on the Gut Microbiota in UC Mice

#### 3.5.1. Bioinformatic and Taxonomic Composition Analysis

**Figure 3:**
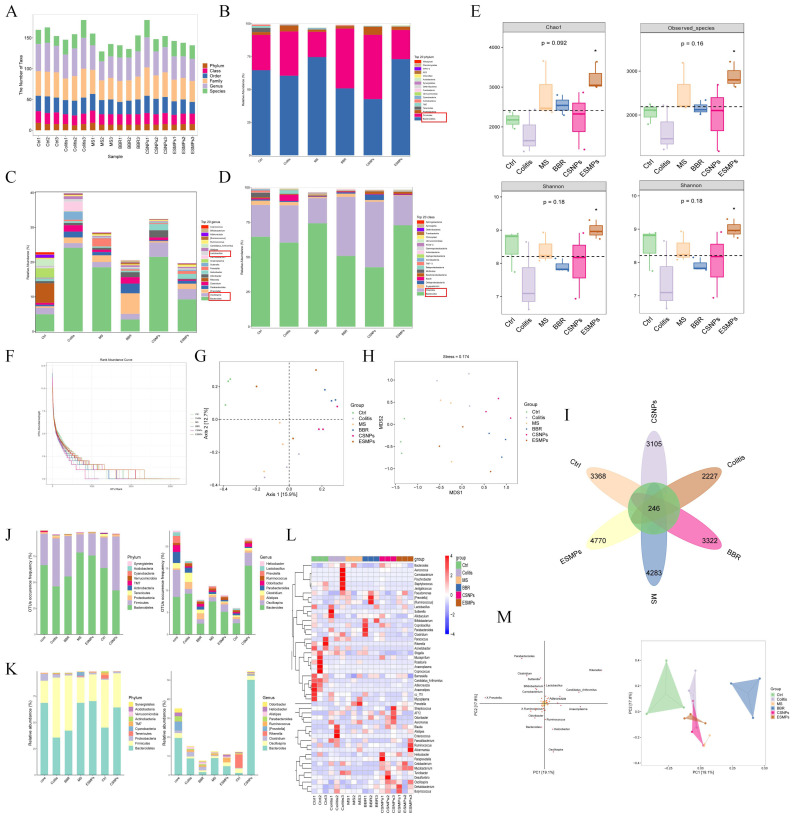
Effects of berberine microparticles on the gut microbiota in UC mice (**A**) Statistical chart of microbial taxonomic units across classification levels (Phylum, Class, Order, Family, Genus, Species). (**B**) Phylum-level taxonomic composition bar chart. (**C**) Genus-level taxonomic composition bar chart. (**D**) Class-level taxonomic composition bar chart. (**E**) Alpha diversity indices (Observed species, Chao1, Shannon, Simpson), * *p* < 0.05. (**F**) Rank-abundance curves illustrating community evenness. (**G**) Two-dimensional ordination plot of Principal Coordinate Analysis (PCoA) (**H**) Non-metric Multidimensional Scaling (NMDS) ordination plot (stress = 0.174). (**I**) Venn diagram of shared and unique ASV/OTUs across groups. (**J**) Bar chart of ASV/OTU counts in distinct Venn diagram regions. (**K**) Bar chart of ASV/OTU abundance in distinct Venn diagram regions. (**L**) Heatmap of genus-level microbial composition. (**M**) Orthogonal Partial Least Squares-Discriminant Analysis (OPLS-DA) loading plot (species contribution) and score plot (sample clustering).

The taxonomic units across six classification levels (Phylum, Class, Order, Family, Genus, Species) are summarized in Figure 3A.

##### Phylum-Level Composition

As shown in Figure 3B, 20 bacterial phyla were identified across 18 samples, with Firmicutes and Bacteroidetes dominating (>90% combined relative abundance). The Firmicutes/Bacteroidetes (F/B) ratio, a marker of dysbiosis, was significantly elevated in the Colitis group compared to the Ctrl group (*p* < 0.01), characterized by reduced Bacteroidetes and increased Firmicutes abundance. Post-treatment, the MS and ES MP groups exhibited restored Bacteroidetes levels and reduced F/B ratios, consistent with clinical IBD remission trends. In contrast, the BBR and CS NP groups showed no significant improvement in F/B ratios, highlighting the superior microbiota-modulating efficacy of colon-targeted BBR-ES MPs.

##### Genus-Level Composition

Figure 3C illustrates genus-level shifts, where Bacteroides and Lactobacillus were markedly enriched in the Colitis group (*p* < 0.01 vs. Ctrl). Therapeutic interventions, particularly ES MPs, normalized these genera to near-Ctrl levels, demonstrating targeted restoration of gut microbial balance.

##### Class-Level Composition

At the class level (Figure 3D), Clostridia abundance surged, while Bacteroidia declined in the Colitis group (*p* < 0.01). Treatment with MS and ES MPs reversed these trends, confirming their ability to ameliorate DSS-induced dysbiosis.

Collectively, berberine modulated the abundance of key phyla, genera, and classes in UC mice, restoring gut microbiota structure and diversity. Colon-targeted BBR-ES MPs exhibited enhanced efficacy, underscoring the importance of site-specific drug delivery in microbiota-driven UC therapy.

#### 3.5.2. The Results of Alpha Diversity Analysis

##### Alpha Diversity Indices

The Observed species and Chao1 indices reflected microbial richness, while the Shannon and Simpson indices indicated community evenness. As shown in Figure 3E, the Colitis group exhibited reduced species richness and evenness compared to the Ctrl group, though the differences were not statistically significant (*p* > 0.05). All treatment groups showed improved alpha diversity compared to the Colitis group, with the ES MP group demonstrating the most significant enhancement (*p* < 0.05).

##### Rank-Abundance Curves

Figure 3F displays rank-abundance curves, where a steeper slope indicates lower community evenness. The Colitis group exhibited steeper curves than the Ctrl group, reflecting dysbiosis. While the CS NP group retained a steep slope, other treatment groups (especially ES MPs) showed flattened curves, indicating restored microbial evenness.

#### 3.5.3. The Results of Beta Diversity Analysis

##### PCoA and NMDS

Principal coordinate analysis (PCoA) and non-metric multidimensional scaling (NMDS) were performed using QIIME to evaluate inter-group microbial dissimilarity (Figure 3G,H). PCoA revealed distinct clustering between the Ctrl and Colitis groups, with treatment groups (particularly ES MP) shifting toward the Ctrl cluster. NMDS (stress value = 0.174, <0.2) corroborated these findings, confirming that berberine interventions restored gut microbiota composition closer to healthy controls.

#### 3.5.4. Differential Species and Biomarker Analysis

##### ASV/OTU Venn Diagram

Figure 3I–K identifies 246 shared OTUs across groups. Phylum- and genus-level analyses aligned with earlier findings: the Colitis group showed elevated Firmicutes and reduced Bacteroidetes (consistent with Section 3.5.1), while Bacteroides and Lactobacillus were overrepresented (Section 3.5.1). Treatment groups, notably ES MP, reversed these trends.

##### Heatmap of Genus-Level Composition

Figure 3L highlights distinct microbial profiles between the Colitis and Ctrl groups. The BBR group partially resembled the Colitis group, whereas the CS NP and ES MP groups exhibited profiles closer to that of the Ctrl, underscoring their therapeutic efficacy.

##### OPLS-DA Analysis

Orthogonal partial least squares-discriminant analysis (OPLS-DA) modeled relationships between microbial abundance and group classification. Figure 3M shows minimal separation among the Ctrl, CS NP, and ES MP groups, indicating similar microbial richness. This confirms that berberine, particularly via colon-targeted MPs, mitigates DSS-induced dysbiosis by restoring microbial abundance, structure, and metabolic balance, thereby alleviating UC pathology.

## 4. Discussion

Ulcerative colitis (UC), a chronic inflammatory bowel disease (IBD), is characterized by mucosal inflammation, abdominal pain, diarrhea, and hematochezia. Its complex etiology and variable treatment responses pose significant clinical challenges. This study developed a dual pH-responsive drug delivery system utilizing berberine-loaded chitosan nanoparticles (BBR-CS NPs) and berberine-embedded microparticles (BBR-ES MPs) to achieve colon-targeted delivery and enhance therapeutic efficacy in a UC murine model. Results demonstrated improved berberine delivery and modulation of gut microbiota, offering a novel strategy for UC management.

The innovation of this study lies in designing a dual-responsive system adapted to the gastrointestinal pH gradient. The pH shifts from the stomach (pH 1.5–3.5) to the small intestine (pH 6.0–7.4) and colon (pH 5.5–7.0), with UC inflammation further acidifying the local microenvironment. BBR-CS NPs and BBR-ES MPs exploit this feature, minimizing drug release in the upper gastrointestinal tract while maximizing delivery at inflamed colonic sites. Compared to free BBR or non-targeted controls, BBR-CS NPs exhibited superior efficacy, including reduced disease activity index (DAI) and alleviated weight loss. These findings align with the critical role of colon targeting in IBD therapy. While prior studies used pH-sensitive polymers (e.g., Eudragit^®^) for targeting, such systems struggle to adapt to UC’s dynamic microenvironment [25]. Our approach combines chitosan’s mucoadhesive properties with berberine’s pharmacological effects, ensuring sustained drug release at inflammatory sites. ANOVA (*p* < 0.05) confirmed significant differences between treatment and control groups, highlighting the system’s superiority.

Berberine’s anti-inflammatory, antioxidant, and antimicrobial effects in UC are well-documented [26]. Targeted delivery via BBR-CS NPs amplified these benefits. Reduced DAI and stabilized body weight in treated mice suggest potent inflammation suppression, likely mediated by downregulating cytokines like TNF-α and IL-6. The enhanced efficacy of BBR-CS NPs over BBR-ES MPs or free BBR implies additional mechanisms, such as improved cellular uptake and prolonged drug retention. Chitosan’s positive charge promotes adhesion to inflamed colonic mucin, elevating local drug concentrations—a critical advantage in UC’s compromised mucosal barrier. The small size of BBR-CS NPs (<200 nm) may enhance epithelial or immune cell endocytosis, whereas BBR-ES MPs rely more on passive diffusion, yielding comparatively weaker effects.

BBR-CS NPs significantly modulated gut microbiota. NMDS analysis (stress value 0.174 < 0.2) revealed distinct microbial clustering between treated, colitis, and control groups, indicating berberine-driven microbial remodeling. Colitis-induced diversity loss was reversed post-treatment, with OTU counts increasing to 246, potentially enriching beneficial taxa (e.g., *Lactobacillus*) and suppressing pathogens (e.g., *Escherichia*), consistent with berberine’s antimicrobial properties (cite). This may support epithelial repair and immune balance via short-chain fatty acid regulation (e.g., butyrate). BBR-ES MPs showed weaker microbiota modulation, underscoring the nano-system’s advantage. While microbial interventions for IBD are emerging [27,28], our approach synergizes targeted drug delivery with microbiota regulation for enhanced outcomes.

Current UC therapies (e.g., aminosalicylates, corticosteroids, anti-TNF agents) are limited by side effects and lack of specificity. BBR-CS NPs, combining natural compound safety with nanotechnology, offer a promising alternative. Unlike budesonide microparticles or mesalazine, they address both inflammation and dysbiosis, bridging gaps in existing therapies and positioning themselves as next-generation candidates. However, the DSS-induced murine model used here did not fully replicate human UC’s chronicity or immune complexity, and interspecies microbiota differences may limit translational relevance. While NMDS and ANOVA confirmed statistical significance, key microbial drivers remain unidentified. BBR-CS NP stability requires optimization, and long-term safety (e.g., hepatorenal toxicity) remains unassessed. Future work should explore interactions between BBR-CS NPs, immunity, and microbial metabolism, with potential applications extended to pH-dependent diseases like Crohn’s or colorectal cancer.

In conclusion, dual pH-responsive BBR-CS NPs and BBR-ES MPs advance UC treatment through targeted delivery, anti-inflammatory action, and microbiota modulation. This study provides new insights into IBD management, demonstrating the potential of nanotechnology-natural compound hybrids and laying the groundwork for future research.

## 5. Conclusions

In this study, we developed for the first time a dual pH- and enzyme-responsive colon-targeted drug delivery system encapsulating berberine (BBR) and elucidated its therapeutic efficacy in ulcerative colitis (UC). Our findings demonstrate that BBR-loaded dual-responsive microparticles (BBR-ES MPs) significantly alleviated dextran sulfate sodium (DSS)-induced UC, as evidenced by reduced pro-inflammatory cytokine levels (IL-1β, IL-6, TNF-α), improved histopathological outcomes, and diminished myeloperoxidase (MPO) activity. Furthermore, alpha and beta diversity analyses revealed that DSS induction disrupted gut microbiota composition, reducing microbial richness and evenness, which were partially restored post-treatment. Differential species analysis highlighted distinct microbial shifts in UC mice, with BBR-ES MPs modulating dysbiosis by recalibrating microbial abundance, restoring structural balance, and ameliorating metabolic dysregulation. This study not only validates the therapeutic potential of BBR-ES MPs for UC but also establishes a foundational framework for advancing cost-effective, precision-targeted berberine-based therapies in inflammatory bowel disease management.

## Figures and Tables

**Table 1:** Disease activity index of mice (DAI).

Score	Loss of Weight (%)	Stool Property	Archorrhagia
0	0	Normal	Normal
1	1~5		
2	6~10	Semi-loose stools	Hyporrhea
3	11~15		
4	>15	Loose stools	Severepostpartumbleeding

**Table 2:** The equations and parameters of the First-order model and Higuchi model.

		First-Order Model			HIGUCHI	
		Q = Qm(1 - e^−kt^)			Q = Kt^1/2^ + b	
	Qm	k	R2	k	b	R2
bbr	93.80078	0.44329	0.96671	6.56734	45.0763	0.35324
bbr-cs nps	89.95636	1.36394	0.99938	4.08993	62.91878	0.17352
bbr-es mps	50,737.76517	6.34 × 10^−5^	0.79152	6.87697	−2.8994	0.79922
bbr-es mps + β-g	−53,502.00682	−8.32 × 10^−5^	0.80291	9.17465	−1.70147	0.82228

## Data Availability

Data will be made available on request.

## Supplementary Materials

The following supporting information can be downloaded at: https://www.mdpi.com/article/10.3390/pharmaceutics17060778/s1, Figure S1: 3D response surface (left) and contour map (right) on complexation particle sizes; Figure S2: 3D response surface (left) and contour map (right) on complexation entrapment efficiency; Table S1: Survey of CS and TPP at different mass ratio (*n* = 3); Table S2: Survey of CS NPs at different chitosan concentrations (*n* = 3); Table S3: Survey of mixing speeds (*n* = 3); Table S4: Survey of different mixing time (*n* = 3); Table S5: Survey of drug loading ratio (*n* = 3); Table S6: Results of central composite design; Table S7: Analysis of variance of size; Table S8: Analysis of variance of encapsulation rate; Table S9: Feed ratio screening result (*n* = 3, $\bar{x}$ ± s); Table S10: Entrapment efficiency and drug loading result (*n* = 3, $\bar{x}$ ± s).

## Abbreviations

The following abbreviations are used in this manuscript:

UC	Ulcerative colitis
BBR-CS NPs	Berberine-loaded chitosan nanoparticles
BBR-ES MPs	Berberine Eudragit S-100 microparticles
