# Supplementary material for: Design and Activity Evaluation of Berberine-Loaded Dual pH and Enzyme-Sensitive Colon-Targeting Microparticles
Source: Pharmaceutics. 2025 Jun 13;17(6):778. doi: 10.3390/pharmaceutics17060778 (PMC12196873; doi:10.3390/pharmaceutics17060778)
Supplement: Supplementary file 1 [file pharmaceutics-17-00778-s001.zip › pharmaceutics-3640269-supplementary.pdf]

pharmaceutics

Supplementary Materials: Design and Activity Evaluation of Berberine-Loaded Dual pH and Enzyme-Sensitive Colon-Targeting Microparticles

Jingqi Sun, Xinlong Chai, Xiwen Zeng, Qingwei Wang, Yanwen Ling, Lihong Wang and Jin Su

Table S1. Survey of CS and TPP at different mass ratio(n=3).

The mass ratio of CS to TPP	Mean grain size(nm)	PDI
2:1	359.5±5.6	1.102±0.057
3:1	165.6±5.1	0.248±0.048
4:1	195.4±4.8	0.339±0.051
5:1	206.0±5.3	0.474±0.049
6:1	210.2±2.5	0.457±0.076

Table S2. Survey of CS NPs at different chitosan concentrations (n=3).

The concentration of CS(mg·mL^{-1})	Mean grain size(nm)	PDI
1	175.4±4.5	0.240±0.041
2	241.4±1.3	0.357±0.016
3	309.4±3.8	0.475±0.034
4	342.6±4.7	0.422±0.059
5	418.6±2.8	0.494±0.071

Table S3. Survey of mixing speeds (n=3).

Mixing speed r·min^{-1})	Mean grain size(nm)	PDI
300	169.6±6.8	0.368±0.031
400	167.8±4.5	0.322±0.028
500	161..2±6.1	0.334±0.029
600	154.6±2.4	0.254±0.034
700	159.2±3.5	0.292±0.051

Table S4. Survey of different mixing time (n=3).

Mixing time （min）	Mean grain size(nm)	PDI
10	152.2±6.4	0.269±0.029
20	147.0±4.6	0.261±0.025
30	163.2±5.2	0.245±0.036
40	166.3±3.8	0.338±0.019
50	180.8±4.5	0.333±0.072

Table S5. Survey of drug loading ratio (n=3).

Drug loading ratio	Mean grain size(nm)	Encapsulation Efficiency (%)
1:1	171.5±4.7	27.8±2.3
2:1	165.6±5.1	55.6±2.9

4:1	156.4±4.8	67.5±2.1
6:1	152.0±5.3	85.3±1.9
8:1	164.2±2.5	79.6±1.5

Table S6. Results of central composite design.

Run	X_1	X_2	X_3	Y_1/nm	Y_2/%
1	3.00	6.00	20.00	156	82.5
2	4.59	7.19	25.95	160.9	61.1
3	4.00	6.00	30.00	146.5	41.9
4	4.00	6.00	10.00	160.8	75.8
5	3.41	7.19	25.95	154.4	51.8
6	3.41	4.81	25.95	147.8	50.4
7	4.00	6.00	20.00	238.1	59.5
8	4.00	6.00	20.00	237.2	60.4
9	3.41	4.81	14.05	157.6	85.4
10	4.00	6.00	20.00	238.4	59.8
11	4.59	7.19	14.05	163.8	68.4
12	4.00	8.00	20.00	172.4	52.4
13	4.00	6.00	20.00	238.6	58.4
14	4.00	6.00	20.00	238.5	57.6
15	4.00	6.00	20.00	237.4	59.4
16	5.00	6.00	20.00	166.1	79.4
17	4.59	4.81	25.95	150.7	47.3
18	4.00	4.00	20.00	168.2	47.9
19	4.59	4.81	14.05	166.5	70.5
20	3.41	7.19	14.05	155.8	72.6

Table S7. Analysis of variance of size.

Source	Sum of Squares	df	Mean Square	F Value	P-F
Model	26952.75	9	2994.75	5163.45	< 0.0001**
A	137.20	1	137.20	236.55	< 0.0001**
B	27.45	1	27.45	47.34	< 0.0001**
C	213.12	1	213.12	367.46	< 0.0001**
AB	0.91	1	0.91	1.57	0.2386
AC	7.03	1	7.03	12.12	0.0059
BC	56.71	1	56.71	97.78	< 0.0001**
A2	10650.59	1	10650.59	18363.40	< 0.0001**
B2	8242.22	1	8242.22	14210.96	< 0.0001**
C2	12799.24	1	12799.24	22068.04	< 0.0001**
Residual	5.80	10	0.58		
Lack of Fit	4.03	5	0.81	2.27	0.1945
Pure Error	1.77	5	0.35		
Cor Total	26958.55	19			

* $p < 0.05$，** $p < 0.01$

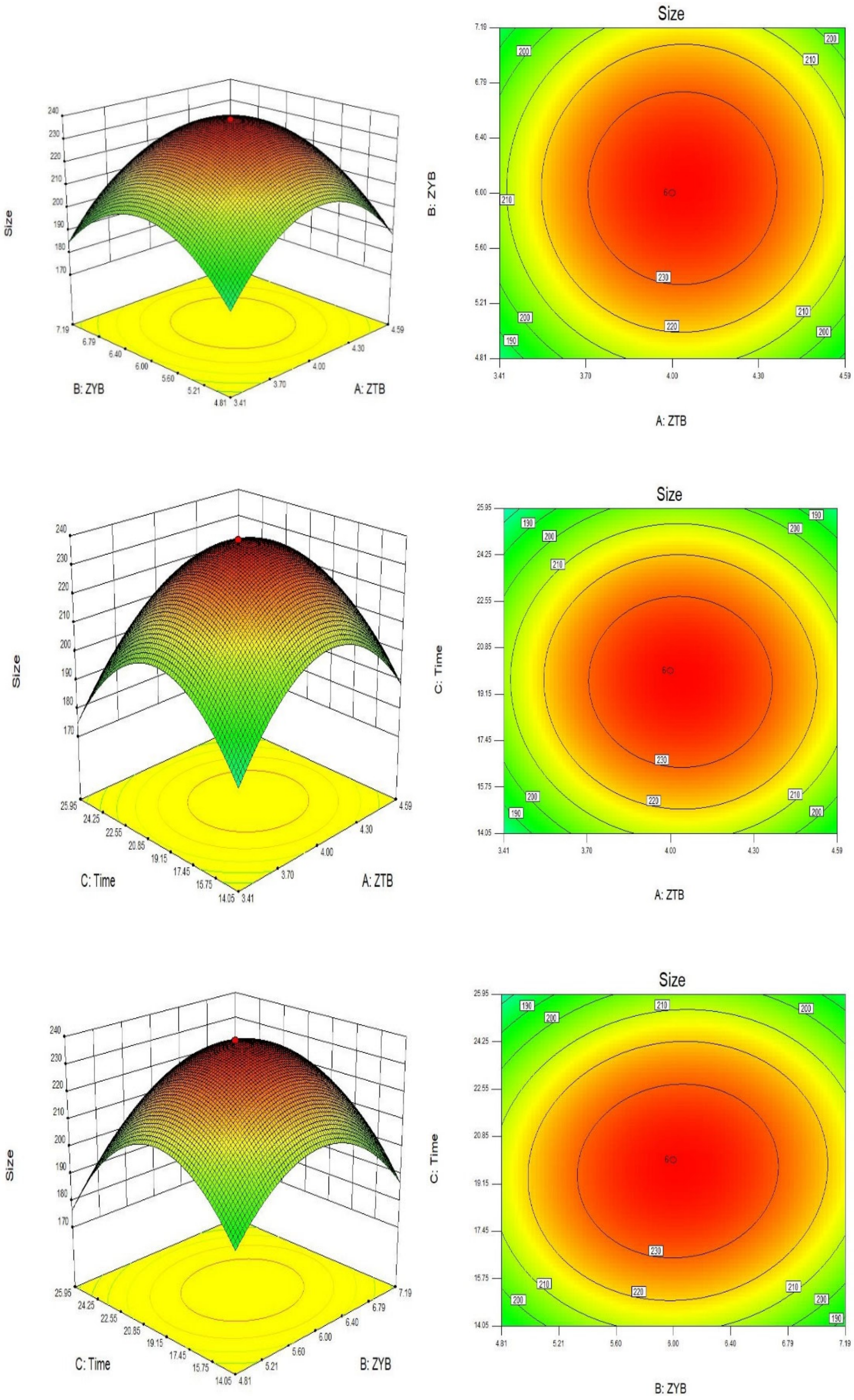

Figure S1. 3D response surface(left) and contour map(right) on complexation particle sizes.

Table S8. Analysis of variance of encapsulation rate.

Source	Sum of Squares	df	Mean Square	F Value	P-F
Model	2875.08	9	319.45	221.94	< 0.0001**
A	24.02	1	24.02	16.69	0.0022*
B	4.53	1	4.53	3.15	0.1064

C	1503.90	1	1503.90	1044.82	< 0.0001**
AB	66.70	1	66.70	46.34	< 0.0001**
AC	80.01	1	80.01	55.59	< 0.0001**
BC	113.25	1	113.25	78.68	< 0.0001**
A2	847.42	1	847.42	588.73	< 0.0001**
B2	149.53	1	149.53	103.88	< 0.0001**
C2	0.30	1	0.30	0.21	0.6556
Residual	14.39	10	1.44		
Lack of Fit	9.27	5	1.85	1.81	0.2660
Pure Error	5.13	5	1.03		
Cor Total	2889.48	19			

* $p < 0.05$，** $p < 0.01$.

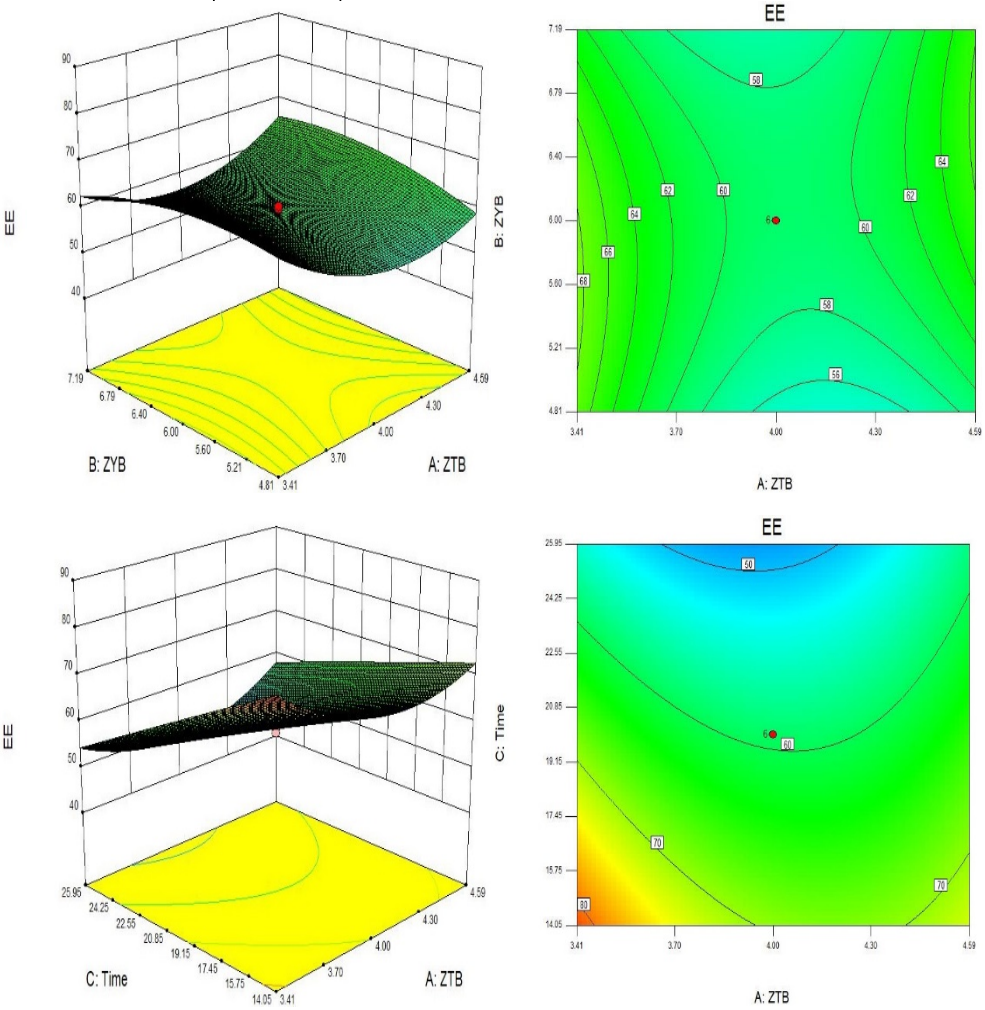

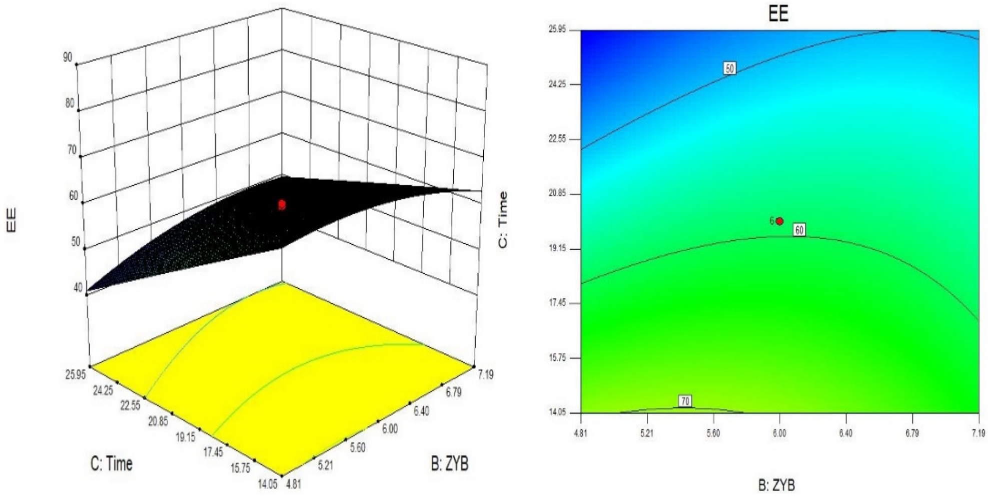

Figure S2. 3D response surface(left) and contour map(right) on complexation entrapment efficiency.

Table S9. Feed ratio screening result (n=3, $\bar{x}$±s).

Rate of charge	Encapsulation Efficiency （%）	Drug loading capacity（%）
1:1	33.32±2.72	3.42±0.62
2:1	59.31±3.14	3.18±0.42
3:1	72.24±3.28	2.56±0.51
4:1	75.14±3.69	1.34±0.48
5:1	49.78±5.67	1.12±0.39

Table S10. Entrapment efficiency and drug loading result（n=3, $\bar{x}$±s）.

Sample	Encapsulation Efficiency （%）	Drug loading capacity（%）
BBR-ES NPs	74.25±2.74	2.56±0.43
